# Effect of tibiofemoral alignment on simulated knee contact forces during gait in mechanically and kinematically aligned total knee arthroplasty patients

**DOI:** 10.1038/s41598-024-78618-6

**Published:** 2024-11-11

**Authors:** Stefanie John, Torm Bierwirth, Dennis Nebel, Ann-Kathrin Einfeldt, Eike Jakubowitz, Lars-René Tücking, Peter Savov, Max Ettinger, Henning Windhagen, Christof Hurschler, Michael Schwarze

**Affiliations:** 1 Laboratory for Biomechanics and Biomaterials, Department of Orthopaedic Surgery, Hannover Medical School, DIAKOVERE Annastift, Anna von Borries Str. 1-7, 30625 Hannover, Germany; 2 Department of Orthopaedic Surgery, Hannover Medical School, DIAKOVERE Annastift, Anna von Borries Str. 1-7, 30625 Hannover, Germany; 3 Department of Orthopaedic and Trauma Surgery, University of Oldenburg, Pius Hospital, Georgstraße 12, 26121 Oldenburg, Germany; 4 Department for Medical Technology, Bremerhaven University of Applied Sciences, An der Karlstadt 8, 27568 Bremerhaven, Germany

**Keywords:** Biomedical engineering, Computational models

## Abstract

The goal of the study was to apply a musculoskeletal knee model that considers individual tibiofemoral alignment (TFA) and to investigate its effect on knee contact force (KCF) during gait in mechanically (MA) and kinematically aligned (KA) total knee arthroplasty (TKA) patients. Total, medial, and lateral KCF was estimated from pre- and postoperative gait data of TKA patients (MA: *n* = 26, KA: *n* = 22). Preoperative KCF was compared between the generic and the adapted model using t-tests and statistical parametric mapping (SPM). The TFA-adapted model was then used to analyze pre- to postoperative differences in MA and KA patients. The factor of TFA increased estimates of KCF during the stance phase and led to higher peak contact forces (3–5%, *p* < 0.05). SPM analyses of pre- to postoperative KCF revealed no significant differences across the gait cycle, however, postoperative peak KCF was significantly increased in both groups (10–18%, *p* < 0.05). No group differences were observed when comparing KCF between MA and KA patients. Integrating TFA into the model led to higher estimations of KCF. Applying the adapted model, pre- to postoperative differences in KCF were the same for both TKA groups suggesting that both alignment techniques had comparable effects on knee loading post-TKA.

## Introduction

Musculoskeletal (MS) models are a powerful tool for gaining insights into internal knee joint forces during motion that can only be measured directly with instrumented implants^1,2^. Based on anthropometric models and 3D gait analysis kinematic and kinetic data, such models use an inverse dynamics approach to calculate joint moments and forces and consider internal contributions of the muscles surrounding and acting on the knee joint to estimate in-vivo knee contact forces (KCF)^3–5^. MS models also allow the differentiation of the knee forces in the medial and lateral knee compartments, which is important when considering frontal knee malalignment^6^. In total knee arthroplasty (TKA), and in particular, in the context of different alignment philosophies, the magnitude and location of in-vivo knee joint loading have become particularly important for assessing patient function and the risk of load-induced implant failure. MS models have thus found use in studies of patients receiving TKA in order to investigate the distribution of KCF during walking^6,7^, in particular with respect to the correlation of KCF with knee pain^5^.

The knowledge of the distribution of internal knee joint forces in the medial and lateral knee joint compartments could also help to evaluate the surgical outcome after TKA. Numerous alignment philosophies are currently being used in TKA surgery. In this context, mechanical and kinematic alignment is of particular importance and predominant application. Traditionally, mechanical alignment (MA) is used to restore a tibiofemoral joint line that is perpendicular to the neutral mechanical leg axis aiming for an equalized load distribution on the implant^8^. Alternatively, kinematic alignment (KA) has been propagated, in which the individual, pre-arthritic knee anatomy is recreated by reconstruction of the native orientation of the tibiofemoral joint line and knee axes^9^. Studies have shown that the clinical outcome measures as well as the objective measures of the knee function of patients operated with KA were at least comparable or somewhat superior to those of patients operated with MA^10– 12^. However, it remains unclear how the different alignment techniques affect knee joint loading. Due to the goal of maintaining the individual tibiofemoral joint line, patients treated with KA-TKA may retain some degree of postoperative varus and valgus alignment, which has been associated with overloading of the medial or lateral compartment in biomechanical studies^13–15^.

The knowledge gained from MS models can provide a deeper insight into the in-vivo forces of the knee joint and help to compare the outcomes of the two different alignment approaches. To accurately simulate knee kinematics and KFC, patient-specific MS tibiofemoral (TF) joint models are required^16,17^. Many different TF joint models exist ranging from simple generic models to very complex patient-specific models. Simplified knee models are idealized as a hinge joint with one degree of freedom (DOF)^16,18,19^. The more detailed knee models represent more realistic knee joint movements by allowing more DOFs in the knee^17,20^. However, these models do not take the contributions of soft tissue, such as ligament forces to knee loading into account. In recent years, several complex patient-specific multibody contact knee models have been developed, which consider the internal motions in the knee as an interaction of acting forces from muscles and ligaments^16,21–23^.

Although many of these complex knee models have been validated using in vivo data of an instrumented knee prosthesis with good results, they have not yet found their way into clinical application^24^. No study has been published to date, that used these complex knee models to examine a large number of patients, which would be important to make valid statements about clinical results and to compare and evaluate surgical procedures. The disadvantage of using very complex subject-specific MS knee models is the time required for modeling and data calculation^25^. The implementation of subject-specific bone geometries from medical images as well as the process of computing muscle, ligament and joint contact forces simultaneously in complex knee models is very time-consuming^22^. To use musculoskeletal models for a wide range of TKA patients and investigate pre- to postoperative differences in KCF, one idea is to utilize a simpler MS knee model and implement one or two subject-specific parameters that are known to be the major influencing factors on internal knee force. Results from studies that developed a complex subject-specific knee model for one TKA patient showed that KCF and the medial-lateral distribution of knee forces are especially influenced by joint morphology and the tibiofemoral alignment (TFA)^26,27^.

Therefore, the goals of this study were to (1) evaluate and quantify the effect of implementing the individual TFA into a computationally efficient and clinically friendly MS model of KCF in TKA patients during gait, and to (2) use this model to compare calculations of KCF between MA -and KA – TKA patients pre- to postoperatively. We hypothesized that implementing the adapted TFA would result in higher calculated TCF and that patients following KA-TKA would present higher medial contact forces postoperatively than MA patients.

## Methods

### Patients

**Fig. 1:**
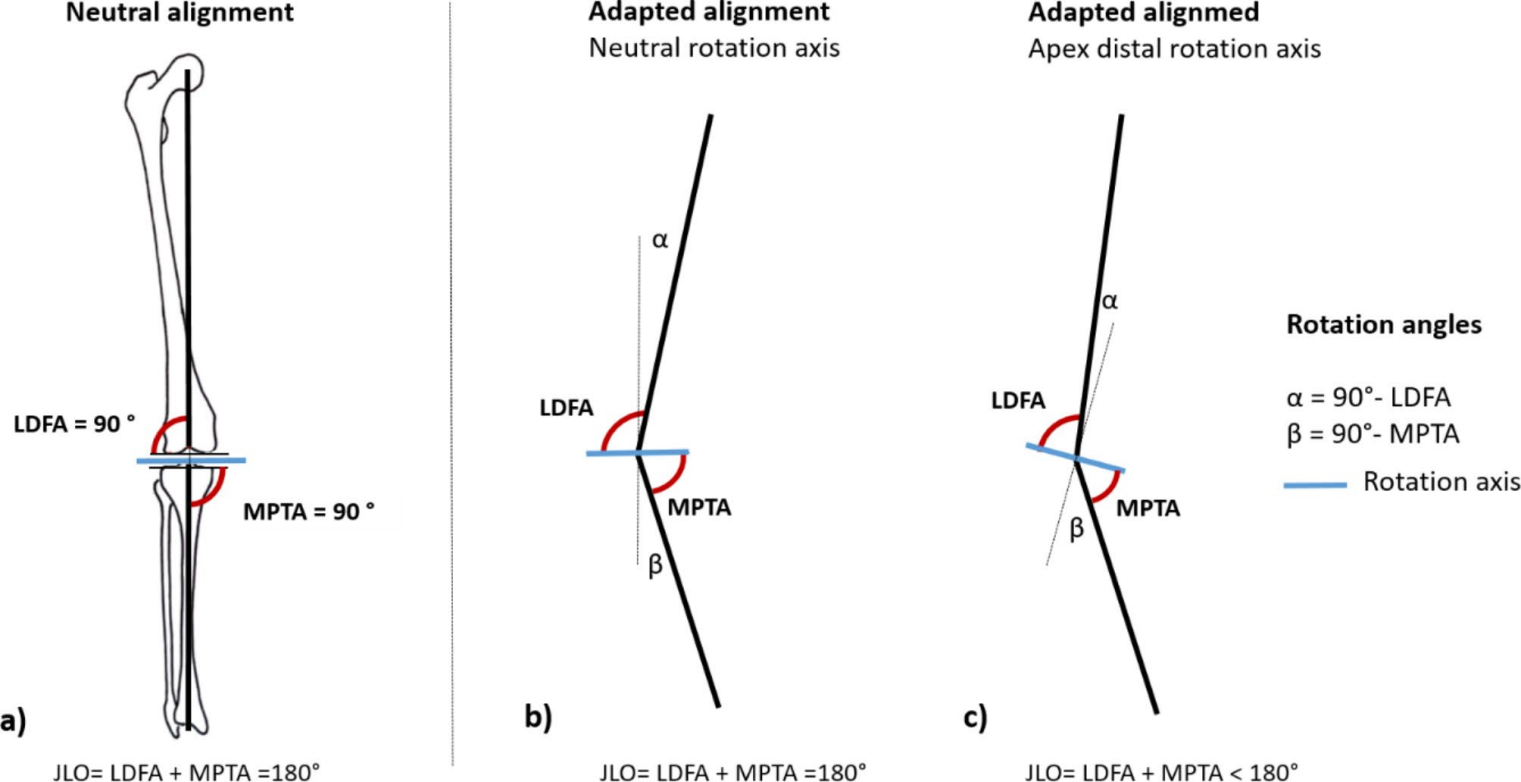
(**a**) Lateral distal femoral angle (LDFA) and medial proximal tibial angle (MPTA) and joint line obliquity (JLO) in neutral alignment model (**b**) Rotation angles α and β used for the adapted alignment model with neutral rotation axis or (**c**) with apex distal rotation axis. Extreme varus angles, which do not occur in practice, were used for clarification.

A total of 46 TKA patients were included in this study with 26 patients assigned to the mechanically aligned (MA) group and 22 patients assigned to the kinematically aligned (KA) group (Table 1). Preoperative inclusion criteria were the indication for primary TKA due to symptomatic osteoarthritis of the knee, a UCLA Activity Score of 4 or greater, and a medial proximal tibial angle (MPTA) between 85° and 90°. Exclusion criteria included a BMI greater than 40 kg/m², a previous osteotomy around the knee, and any previous infection or inflammatory diseases. All patients underwent primary MA- or KA- aligned TKA and received the total knee implant GMK Sphere (Medacta International, Switzerland). A 3D gait analysis was performed before (pre), and one year after surgery (post) using a 12-camera Vicon system (6x MX-F20, 6x MX-F40, VICON Motion System Ltd, Oxford, UK) and two AMTI force plates (model BP400600, AMTI, Watertown, MA, USA) embedded in the floor. Kinematic and kinetic data was collected during level walking and at a self-selected walking speed using the Plug-in Gait (PIG) lower body marker set. The Oxford Knee Score (OKS)^28^ and the Knee Society Score (KSS)^29^ were used as clinical outcome measures to assess pain and function from the patient’s perspective before and after TKA. Radiographic measurements were also taken pre- and postoperatively, including the mechanical hip-knee angle (mHKA), joint line obliquity (JLO), medial proximal tibial angle (MPTA), and lateral distal femoral angle (LDFA). The radiological parameters were determined according to MacDessie et al.^30^ (Fig. 1a). The project was approved by the local ethics committee of Hannover Medical School and was registered in the German Registry of Clinical Trials under the ID DRKS00024567. Written informed consent was obtained from all patients to participate in the project, and all methods were performed in accordance with the Declaration of Helsinki.

## Musculoskeletal model and implementation of TFA

**Fig. 2:**
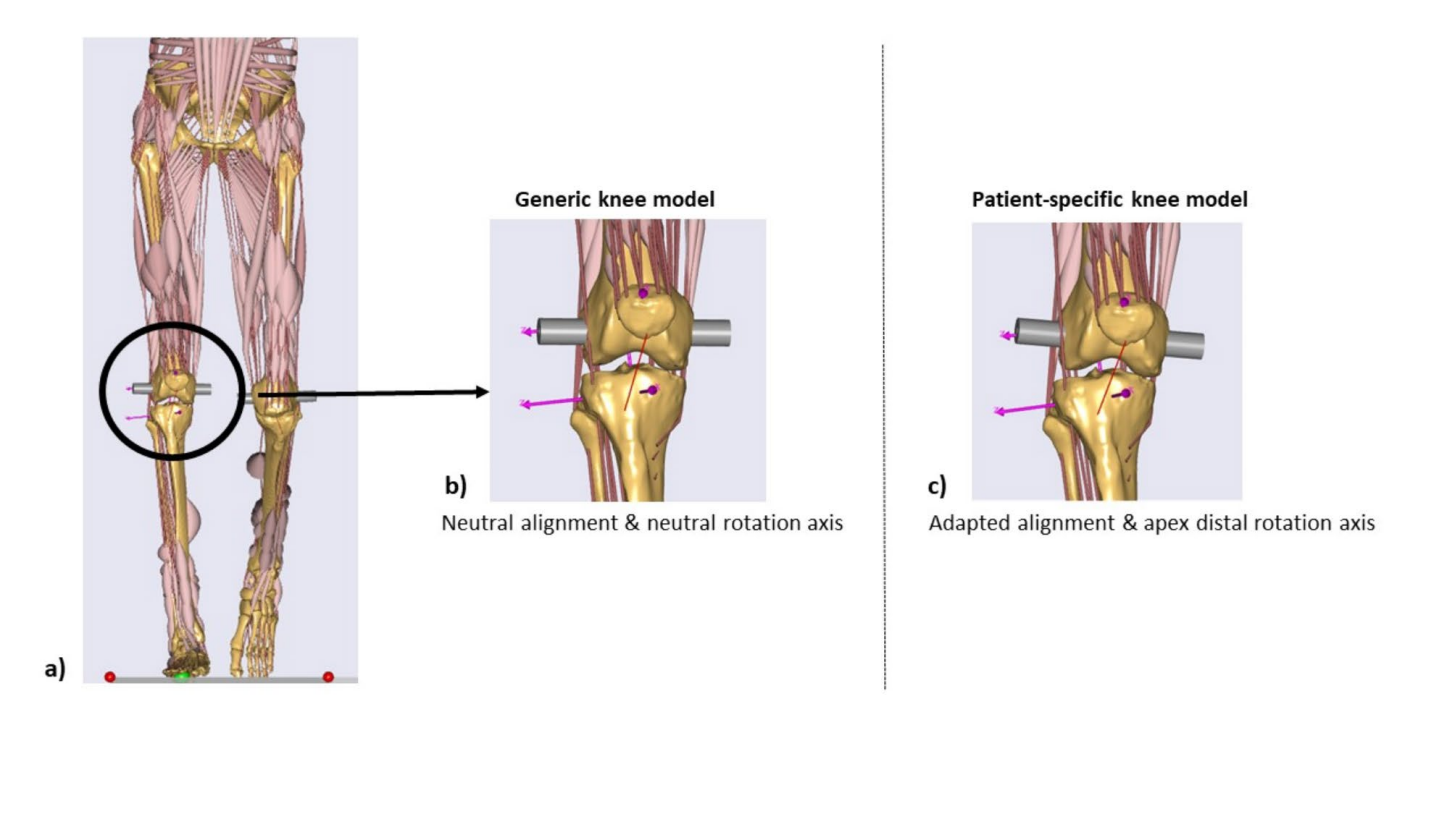
(**a**) MS model of the lower extremities during gait (**b**) Generic knee model with a neutral TFA alignment and neutral rotation axis represented by the grey cylinder (**c**) Patient-specific knee model with the adapted alignment (Femoral valgus = 3°, Tibial varus = 6°) and an apex distal rotation axis (grey cylinder). These models were created in the AnyBody Modeling System Version 7.4.

The Twente Lower Extremity Model v.2.1 (TLEM 2.1) was extracted from the AnyBody Managed Model Repository (AMMR version 1.5.1, AnyBody Technology A/S, Aalborg, Denmark) and served as the basis for the adapted model. It is based on detailed anatomical data produced from a cadaver study conducted by the University of Twente and further refined by De Pieri et al.^31,32^. The upper body was simplified to consist of the lumbar region, trunk, neck and head. The lower extremities were depicted in more detail: each leg contained 55 muscle actuators, divided into 169 elements in accordance with the original TLEM dataset^31^ (Fig. 2a). The muscle elements were represented using a Hill muscle model. Eleven bone segments represented the respective lower limbs, including the pelvis, femur, patella, shank, talus and foot. In the base model, the hip joints are modeled as a ball-and-socket joint with 3 DOFs while the knee, the talocrural, and subtalar joints are assumed hinge joints with 1 DOF. For a more realistic representation of the knee joint, the generic hinge joint was replaced by the AnyKnee model developed and validated by Dzialo et al.^17^ (Fig. 2b).

This knee model includes a scalable tibiofemoral joint with a moving joint axis. The joint axis movement is represented as a function of the tibiofemoral flexion, enabling the simulation of secondary kinematics and therefore more realistic joint kinematics. This model is computationally efficient and allows the evaluation of the knee kinematics within the respective medial and lateral compartments^17^. Individual models were scaled according to the height and mass of each subject. The individual TFA was implemented by using rotation matrices on the anatomical frames of the tibia and femur (Fig. 2c). The amount of rotation applied for both segments depended on the patient-specific femoral varus/valgus (α) and tibial varus/valgus angles (β) and were determined as follows: α = 90°- LDFA where a positive value indicated femoral valgus and a negative value indicated a femoral varus and β = 90°- MPTA where a positive value indicated tibial varus and a negative value indicated tibial valgus. For better clarity on the rotation angles used, a simplified schematic model of the MS model is shown in Fig. 1b, c. In Fig. 1b the rotation angles for a neutral rotation axis are displayed, representing a horizontal joint line, which is achieved only when the LDFA and MPTA sum to 180°. However, most knee types have an apex-distal joint line, where the joint line slopes medially. The rotation angles for this apex-distal alignment are illustrated in Fig. 1c.

## Data processing

The c3d files created during gait data processing with the kinetic and kinematic data served as the basis for the musculoskeletal modeling. To examine the influence of the implementation of an individual TFA on KCF, the inverse dynamic analysis was conducted twice: one time with a neutral TFA from the generic model and one time with the adapted TFA for the patient-specific model. For the comparison of pre-and postoperative knee forces and comparisons between the MA and the KA group, knee forces were only simulated with the adapted TFA model. Only the simulated data of the affected/TKA-treated side of the patients were considered and further analyzed with Matlab R2021a (The MathWorks Inc., Massachusetts, USA). The dynamic total contact force (TCF) in the knee, the percentage acting on medial and lateral knee compartments, as well as the medial contact force (MCF) and the lateral contact force (LCF) were computed and normalized to the body weight. The data are reported over the gait cycle and normalized to 100%.

### Statistical analysis

Statistical analyses were conducted using SPSS 27.0 (IBM Corporation, New York, NY, USA) and statistical parametric mapping (SPM) (version M.0.4.5) with a significance level set to *p* ≤ 0.05. Paired t-tests were performed to investigate the difference between simulated knee forces between the MS models with neutral and adapted TFA. Additionally, root mean square errors (RMSE) were calculated for the mean TCF, MCF, and LCF between the two models.

Paired t-tests were computed to examine the changes in knee contact forces from pre-to postoperative conditions in MA and KA patients. Unpaired t-tests were used to compare variables and knee contact forces between MA and KA patients. For all variables, only the stance phase of the gait cycle (0–60%) was investigated for statistical significance. The effect sizes for the t-tests were determined using Cohen’s d, where d = 0.2 represents a small effect, d = 0.5 a medium effect and d = 0.8 a large effect (Cohen, 1988).

## Results

### Patients

Preoperatively, there were no significant differences between the MA and KA groups in terms of anthropometric and radiographic parameters, as well as clinical scores (*p* > 0.05) (Table 1). Postoperatively, significant group differences with large effect sizes were seen in the radiographic parameters JLO, MPTA and LDFA reflecting the characteristic outcome of mechanical and kinematic alignment. Clinical scores did not reveal any significant postoperative group differences in perceived pain and function between MA and KA patients.

When comparing preoperative to postoperative clinical scores, both TKA alignment groups showed a significant increase in OKS and KSS Function Scores with large effect sizes (*p* < 0.01, d > 1.79). Regarding radiographic parameters, several significant differences from the pre-to postoperative condition were observed for the MA group. JLO, mHKA, MPTA and LDFA were significantly increased after surgery (*p* < 0.01, d > 0.80). For KA patients, significant postoperative differences were only seen in mHKA and walking speed (*p* < 0.01, d = 1.30; *p* < 0.01, d = 0.83).

**Table 1:** Anthropometric and pre-and postoperative radiographic parameters and clinical scores in MA and KA patients.

	MA (*n* = 26)	KA (*n* = 22)	*p*	Cohen’s d
Pre-op.				
Age [yrs.]	64.2 ± 11.5	65.9 ± 10.7	0.60	0.15
Height [cm]	171.0 ± 9.3	176.5 ± 9.3	0.05	0.59
Mass [kg]	85.8 ± 12.5	88.0 ± 15.8	0.60	0.15
mHKA [°]	5.1 ± 3.4	5.8 ± 2.5	0.44	0.23
JLO [°]	173.5 ± 3.1	173.5 ± 2.9	0.96	0.02
MPTA [°]	86.1 ± 2.1	85.7 ± 1.9	0.46	0.22
LDFA [°]	87.4 ± 2.2	87.8 ± 1.6	0.49	0.20
OKS (0–40)	23.4 ± 5.9	24.7 ± 6.7	0.46	0.22
KSS Function Score (0-100)	43.8 ± 8.1	46.6 ± 12.9	0.42	0.27
Walking speed [m/s]	1.2 ± 0.2	1.2 ± 0.2	0.93	0.03
Post-op.				
mHKA [°]	2.4 ± 2.0*	1.3 ± 2.8*	0.13	0.45
JLO [°]	178.4 ± 2.1*	173.3 ± 3.6	**< 0.01**	**1.73**
MPTA [°]	88.2 ± 1.7*	86.4 ± 2.1	**< 0.01**	**0.95**
LDFA [°]	90.1 ± 2.0*	86.9 ± 2.6	**< 0.01**	**1.41**
OKS (0–40)	36.4 ± 7.8*	40.3 ± 6.9*	0.08	0.52
KSS Function Score (0-100)	73.3 ± 17.2*	81.4 ± 17.6*	0.16	0.47
Walking speed [m/s]	1.23 ± 0.16	1.28 ± 0.17*	0.30	0.31

*mHKA* mechanical hip knee angle, *JLO* joint line obliquity, *MPTA* medial proximal tibial angle, *LDFA* lateral distal femoral angle, *OKS* Oxford Knee Score, *KSS* Knee Society Score.

Significant differences between MA and KA patients are marked in bold.

The asterisk (*) indicates a significant difference in the postoperative condition compared to the preoperative condition (*p* < 0.05).

## Preoperative knee forces with and without an adapted TFA

**Fig. 3:**
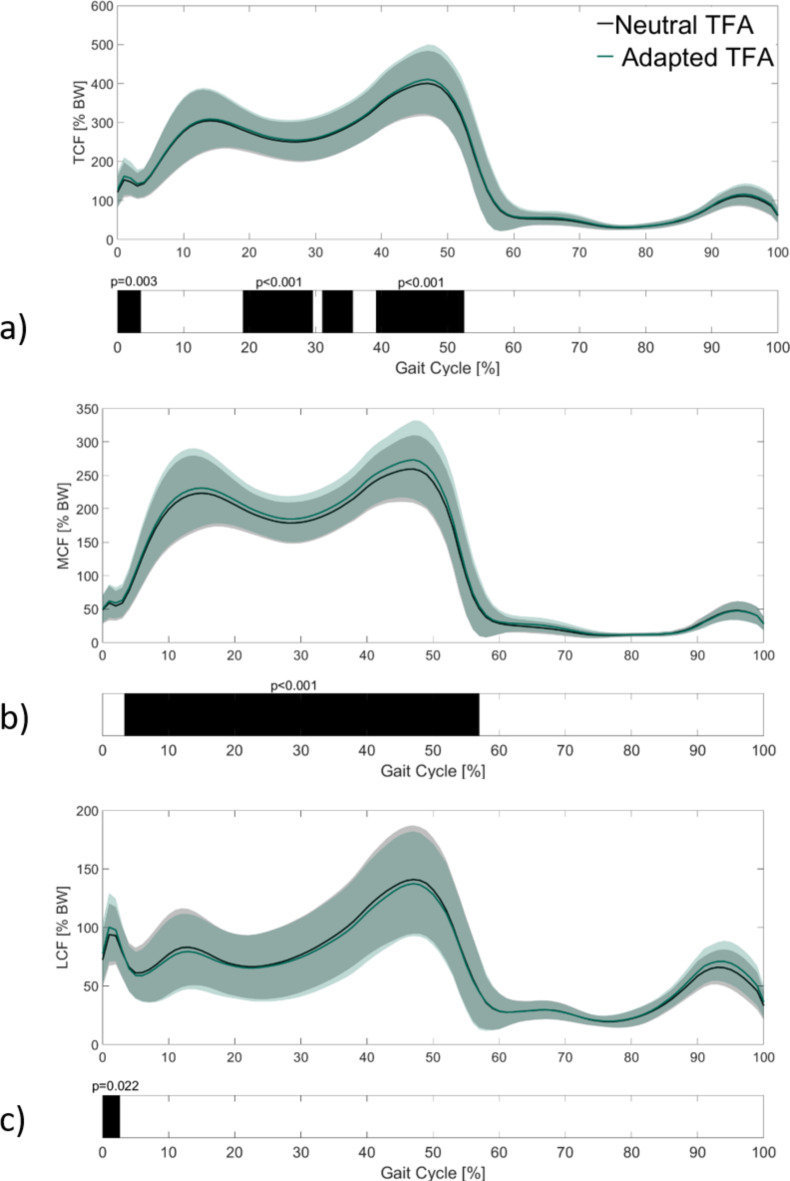
(**a**) Mean and standard deviation of preoperative total knee contact force, (**b**) medial knee contact force, and (**c**) lateral knee contact force with the neutral and the adapted TFA. Significant intervals are indicated by the black bars.

Since there were no significant differences in radiographic parameters in the preoperative condition, the MA and KA patients were combined as one group to investigate the factor of adapted TFA on KCF. Integrating individual TFA led to significantly increased mean contact forces of TCF and MCF over the gait cycle (Fig. 1). TCF was in particular significantly increased around the second peak of the stance phase (39–52%, *p* < 0.01) (Fig. 3a). Medial contact force (MCF) was affected noticeably with significantly increased values throughout the entire stance phase (3–57%, *p* < 0.01) (Fig. 3b). RMSE values were 4.7% for TCF and 6.3% for MCF. Significant differences in lateral contact force (LCF) were only present at the very beginning of the gait cycle (Fig. 3c) with an overall RMSE of 2.9% between models.

The analysis of medial-lateral load distribution revealed that the adapted TFA models resulted in a significantly higher percentage of TCF on the medial side. Accordingly, the percentage of mean lateral load was lower than the percentage of lateral load computed with the neutral TFA (Table 2). The calculated effect sizes showed a small effect for both variables. Concerning the peak knee forces, the adapted TFA led to a significant increase in peak TCF and peak MCF. The relative differences amounted to 3 to 5% and showed a large effect size (Table 2). Peak LCF was not significantly affected.

**Table 2:** Preoperative peak knee contact forces as well as mean load percentage simulated with and without adapted TFA.

Pre-op.	Neutral TFA	Adapted TFA	Relative Diff. (%)	*p*	Cohen’s d
Peak values					
Total contact force [% BW]	419.3 ± 82.4	430.7 ± 87.8	**2.7**	**< 0.01**	**0.80**
Medial contact force [% BW]	275.8 ± 49.3	290.5 ± 57.9	**5.2**	**< 0.01**	**0.90**
Lateral contact force [% BW]	151.3 ± 45.4	149.3 ± 44.0	1.3	0.34	0.14
Mean load percentage					
Medial load [%]	65.6 ± 5.9	66.9 ± 6.6	**2**	**0.01**	**0.37**
Lateral load [%]	34.4 ± 5.9	33.1 ± 6.6	**3.9**	**0.01**	**0.37**

*TFA* Tibiofemoral alignment.

Significant values are marked in bold.

## Pre- and postoperative knee forces in MA and KA patients

**Fig. 4:**
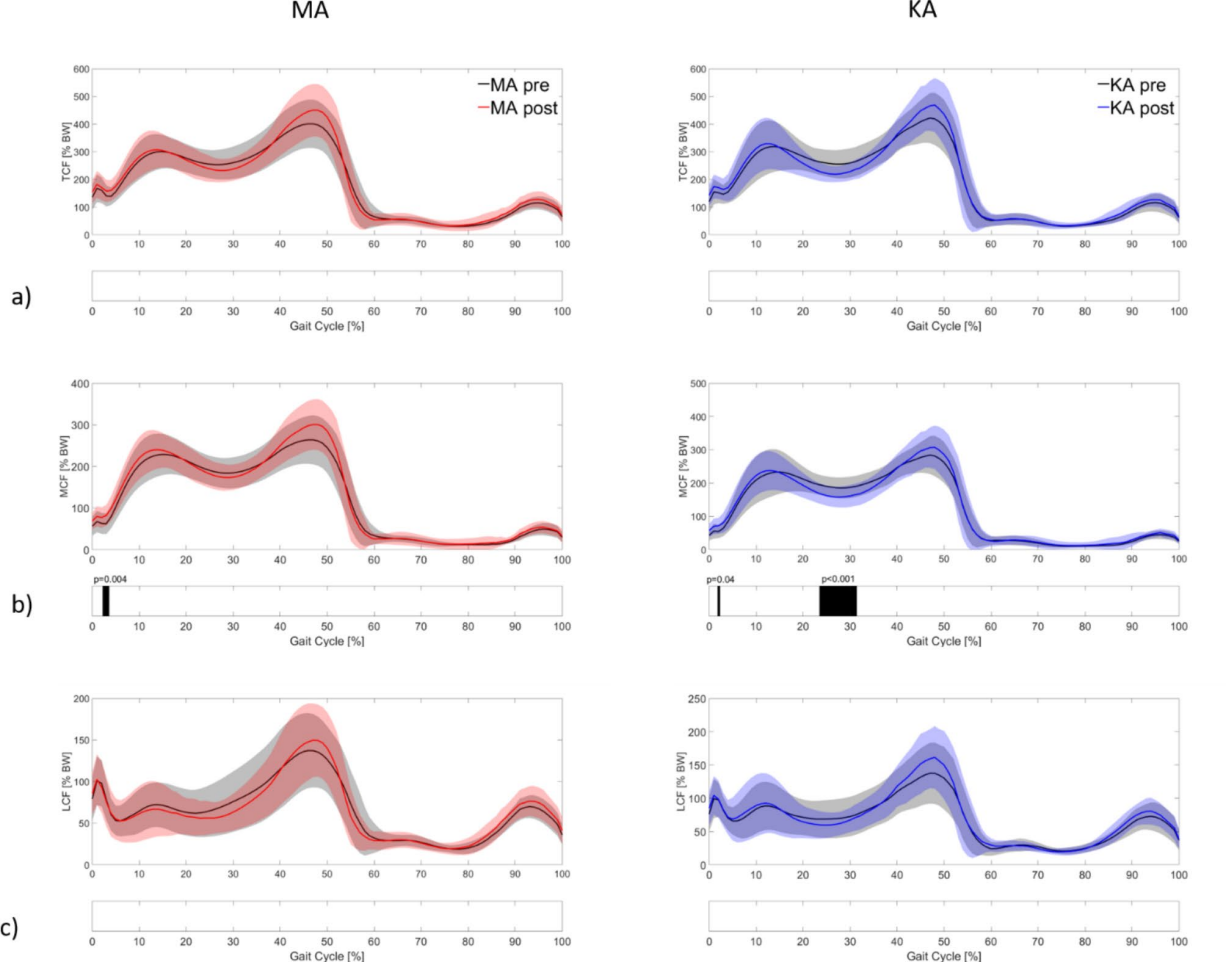
(**a**) Pre and post-operative total knee contact force (TCF), (**b**) Medial contact force (MCF), and (**c**) Lateral contact force (LCF) for MA and KA patients respectively.

Comparing pre- and postoperative KCF with the adapted MS model over the gait cycle, the curves show slightly different courses between the two measurement points (Fig. 4). For MA patients, the mean curves for TCF and MCF indicate postoperatively slightly reduced knee forces in the midstance phase whereas values around the second peak seem to be increased. However, SPM analyses did not reveal any significant differences between the pre- and postoperative conditions. For KA patients, similar results were observed. A significant pre- to postoperative difference, however, was observed for the MCF. MCF was postoperatively reduced between 23 and 31% of the gait cycle (*p* < 0.01) (Fig. 4b). Concerning LCF, the pre- to postoperative joint loading was comparable for both the MA and the KA patients.

**Fig. 5:**
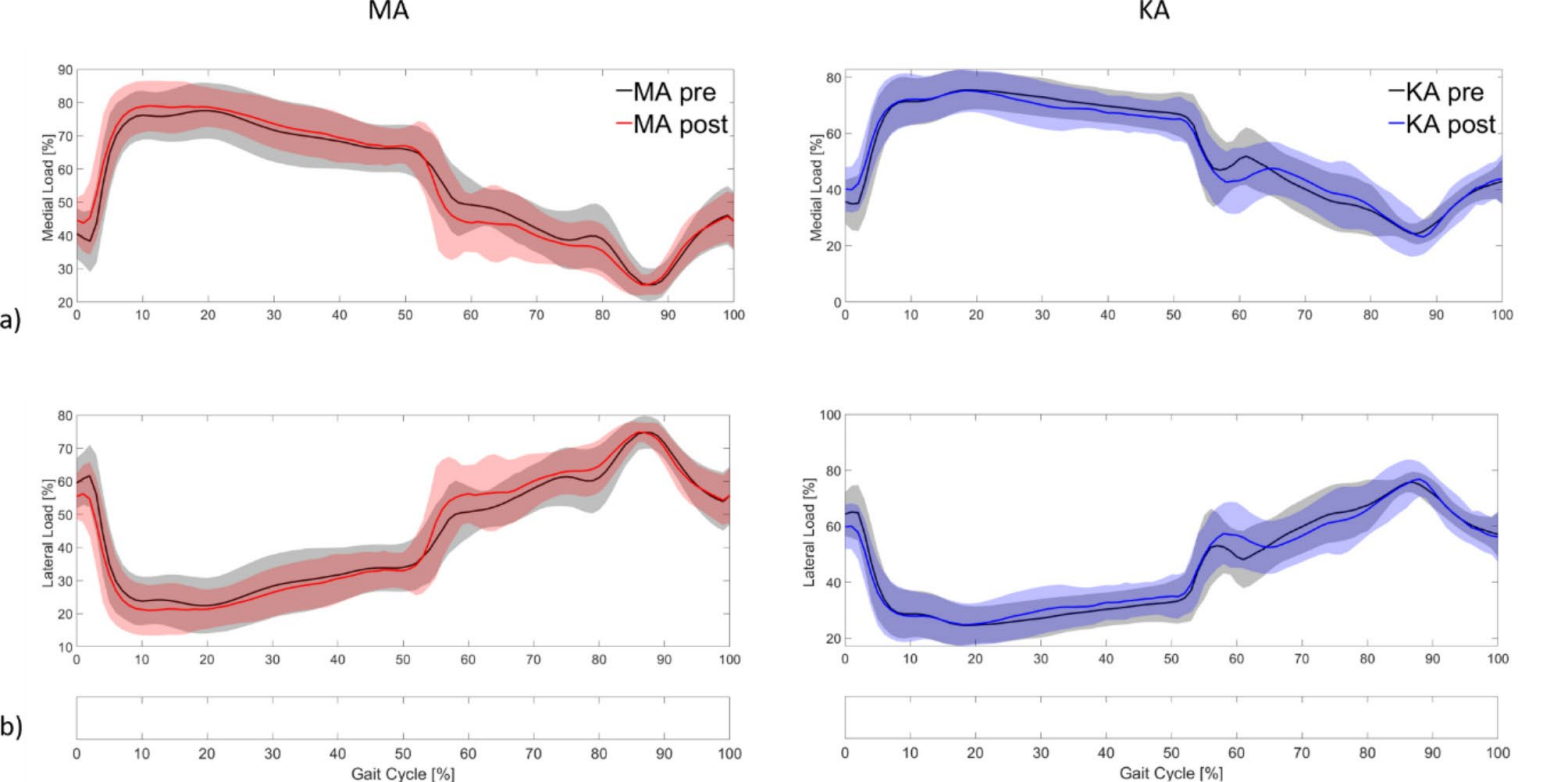
Pre-and postoperative medial (**a**) and lateral (**b**) load distribution over the gait cycle.

SPM group analyses between MA and KA patients for TCF, MCF and LCF also did not show any significant difference for the preoperative or the postoperative condition. Concerning the medial-lateral load distribution, no significant differences from pre- to postoperative conditions could be detected for either MA or KA patients (Fig. 5).

While the SPM analysis of the curves did not reveal any significant differences in the MA patients, peak values for TCF and MCF were significantly higher in the post- than preoperative condition (Table 3). Peak TCF increased by 11% (*p* = 0.03) and peak MCF increased by 10% (*p* = 0.04). Cohen’s d indicated a small effect for both values. The peak LCF remained the same postoperatively. For KA patients, significantly increased peak values were seen for TCF and LCF with relative differences of 12 and 18% compared to the preoperative condition (*p* < 0.01). A medium effect size was observed for both parameters.

When comparing peak KCF between the MA and KA groups, the KA group showed slightly higher contact forces, particularly in the postoperative condition with relative differences of 5 to 8%. However, none of these differences were statistically significant (*p* > 0.05) (Table 3).

**Table 3:** Pre-and postoperative peak knee contact forces of MA and KA patients.

	Pre-op.	Post-op.	Relative Diff. (%)	*p*	Cohen’s d
MA					
Total contact force [% BW]	421.9 ± 88.9	472.6 ± 96.2	**11**	**0.03**	**0.47**
Medial contact force [% BW]	282.1 ± 60.3	313.6 ± 60.1	**10**	**0.04**	**0.41**
Lateral contact force [% BW]	150.9 ± 44.2	162.9 ± 43.9	8%	0.23	0.24
KA					
Total contact force [% BW]	441.1 ± 87.3	498.9 ± 91.3	**12**	**< 0.01**	**0.66**
Medial contact force [% BW]	300.3 ± 54.6	326.1 ± 61.2	8	0.09	0.38
Lateral contact force [% BW]	147.4 ± 44.6	176.9 ± 45.2	**18**	**< 0.01**	**0.71**

*MA* Mechanical alignment, *KA* Kinematical alignment.

## Discussion

The implementation of the individual TFA into the MS model altered the simulated knee contact forces for TKA patients during walking. The TCF and MCF were particularly affected during the stance phase of the gait cycle with increased knee contact forces observed. This confirmed the first hypothesis we investigated.

Using the adapted TFA MS model, an increase in MCF was expected, as varus knee alignment, as it was present in the patients studied, has been shown to alter the medial-lateral contact force distribution. Van Rossom et al.^4^ examined musculoskeletal models with different knee joint malalignments in the frontal plane and reported that medial peak contact forces increased significantly with varus alignment from 3° onwards. Saliba et al.^33^ showed in their MS simulation study that with each degree of varus perturbation, the peak MCF during walking increased by 5 to 6% BW in bothhealthy individuals and patients with knee osteoarthritis. Smith et al.^27^ investigated the influence of frontal TKA component alignment on knee contact force and showed that a 4-degree varus rotation in component alignment resulted in a 17% change in he peak MCF. All these studies used the HKA to define the varus knee alignment and rotated the femur and tibia by an equal amount in the coronal plane. This procedure, however, is based on the assumption of a horizontal joint line, which only occurs in 20 to 30% of the knes in a healthy and arthritic population^30^. Most knee types show an apex-distal joint line obliquity (JLO < 177°)^30^, which was also the case in the majority of patients examined in this study (Table 1). In order to consider the correct JLO and thus correct TFA of the patients, the femur and tibia were rotated by their specific values in this study (Fig. 2b, c). Ro et al.^23^ showed in their study that the same rotation angle of the femur and tibia in the coronal plane affected the knee contact forces differently, which underlines the importance of differentiating between specific tibial and femoral rotations.

Based on the findings of previous studies and based on the results of this study, it can be concluded that it is relevant to consider individual knee alignment to reduce errors in the calculation of KCF. Therefore, the MS model with the adapted TFA was used to investigate changes in KCF between the pre- and postoperative conditions for MA and KA patients. The pre- to postoperative analyses of the knee forces over the gait cycle showed only marginal significant changes in TCF, MCF, LCF and medial-load distribution in the knee between the two measurement points. For KA patients, the medial load was significantly reduced around the midstance phase during the gait cycle postoperatively. Analysis of the peak values revealed a postoperative increase in TCF and MCF in MA patients, while a significantly increased peak TCF and peak LCF was found in KA patients (Table 3).

To date, no study has used MS models to investigate the difference between pre-and postoperative knee contact forces in a larger cohort of TKA patients. Only one other study has investigated the change in TF contact force distribution before and after TKA in two patients with varus alignment. Du et al.^34^ used a complex finite element method (FEM) knee model based on subject-specific bone and ligament geometries derived from MR and CT images as well as implant component implementation and alignment for the post-TKA knee joint model. The authors showed that six months after mechanically aligned TKA, the load on the medial tibial plateau was reduced in static stance and during walking, which could not be reproduced in this study. To evaluate the effect of TKA on medial-lateral force distribution in a large number of patients, most studies have used the knee adduction moment (KAM) from the 3D gait analysis as a surrogate measure of medial contact knee load^11^. Results from 3D gait analyses have shown a decrease in peak knee adduction moment in patients following TKA^35,36^. In this study, however, the peak medial contact force did not show a reduction postoperatively in either the MA or the KA group. Peak KCF were all increased in the postoperative condition. This is most likely attributed to the significantly increased walking speed observed in the postoperative gait analysis (Table 1). Higher walking speed directly influences ground reaction forces, a key factor affecting knee contact forces^35^. Increased peak knee contact forces typically raise clinical concerns, as higher contact forces are linked to increased stress on prosthetic components, potentially raising the risk of wear^14^. However, postoperative increase in KCF may also indicate improved functional capacity, as evidenced by the higher walking speeds observed in the patients postoperatively. This interpretation aligns with the significant improvements in clinical scores seen for both the MA and the KA group (Table 1).

Comparing the knee contact forces between the MA and KA group, no significant group differences were seen in mean TCF, MCF and LCF at any part of the gait cycle. Also, the comparison of peak knee contact forces between the MA and the KA group did not show any significant group differences (Table 3) resulting in the rejection of the second hypothesis we investigated. Postoperative varus alignment and an apex distal JLO, which is likely to occur in KA patients, have previously been associated with varus overload. Studies suggested that patients treated with the KA approach show higher medial contact forces than patients treated with the MA approach^13,14^. This could not be confirmed in this study. However, a recent study comparing KAMs between KA and MA patients reported significantly lower peak KAMs in KA patients than in MA patients^35^. They argued that the oblique joint line is aligned parallel to the ground when walking, which alleviates some of the negative effects of varus alignment on medial overload. Although the subject-specific JLO was considered in the simulations in this study, the results did not show any significant group differences in KCF between alignment groups. One reason that no differences were found may be the heterogeneity in radiologic parameters within both the MA and KA groups. Even if indicated as MA or KA group, the postoperative JLO, MPTA and LDFA varied considerably within both groups. Analyzing subgroups based on the CPAK (coronal plane alignment of the knee) classification that clearly differentiates 9 different knee phenotypes based on TF alignment and JLO^30^ may help to find differences in pre-to-postoperative knee contact forces between the two alignment groups. Additionally, the rather simple MS knee model used in this study may have contributed to this outcome. Although the knee model was improved by implementing an individualized TFA, this one subject-specific parameter may not be sensitive enough to detect differences in these particular patient groups and more complex personalized knee models have to be applied. Another possible explanation for the lack of observed differences between the MA and KA group could be related to the clinical scores. No significant differences in perceived function or pain, which might have influenced gait biomechanics and, consequently, knee contact forces, were found between the two alignment groups.

Some limitations must be considered when interpreting the results of this study. The primary limitation is the simplicity of the MS knee model used. Unlike more complex knee models, this model does not represent certain important anatomical structures, such as ligaments. This simplification may affect the accuracy of predicted knee contact forces and joint kinematics, as ligaments play a crucial role in stabilizing the knee and regulating knee movement. Incorporating ligament behavior could decrease the predicted knee contact forces by redistributing some of the forces to the ligaments, especially in cases where ligament laxity or ligament tightness might be a factor. This could be especially important for the evaluation of the kinematic alignment approach, for which the goal is not only to restore the individual TFA but also to reconstruct the prearthritic JLO and thus the natural ligament tension. However, accurately modeling ligaments presents significant challenges due to their nonlinear mechanical properties, as well as the requirement for detailed imaging, such as MRIs, to determine precise attachment points. Another important factor for future models to consider is the contact forces induced by the femoral and tibial implant components, which were not addressed in this study. Integrating these forces could provide a more comprehensive understanding of joint mechanics and should be accounted for in future research.

However, compared to previously published simulation studies in TKA patients, this study is the first to present MS simulation results of knee contact forces in a larger sample size than typically reported. Implementing the factor of individual TFA in the MS knee model increased the simulated KCF. When comparing postoperative KCF between the TKA alignment groups using the adapted MS knee model, no significant group differences were observed. Importantly, there was no significant increase in medial contact forces in KA patients compared to MA patients, which is considered a potential concern associated with the KA alignment technique. Given the similar increases in peak knee contact forces in both alignment groups, the findings suggest that for the specific patient group studied, both alignment techniques had comparable effects on knee loading post-TKA. This was also reflected in the clinical scores, as both TKA groups demonstrated similar improvements in perceived function and pain.

While the MS model we applied already represents an improvement over the generic MS model normally used, further refinements are warranted. Since only one patient-specific factor for the individualization of the MS knee model was considered in this study, future studies may include further subject-specific parameters but remain time-efficient to be able to be used in larger patient groups and thus be suitable for clinical applications.

## Data Availability

The datasets generated and analyzed during the current study are available from the corresponding author upon reasonable request.
